# Efficacy and Safety of Infliximab in HLA-B27-associated Ocular Inflammation Refractory or Intolerant to Conventional Immunomodulatory Therapy

**DOI:** 10.18502/jovr.v15i4.7786

**Published:** 2020-10-25

**Authors:** Asima Bajwa, Arash Maleki, Abhishek R Payal, Adriana Fandiño, María Inés Menéndez Padrón, Marisa Walsh, C Stephen Foster

**Affiliations:** ^1^Massachusetts Eye Research and Surgery Institution, Waltham, Massachusetts; ^2^Harvard Medical School, Boston, Massachusetts; ^3^Ocular Immunology and Uveitis Foundation, Waltham, Massachusetts; ^4^University of Pennsylvania, Scheie Eye Institute, Philadelphia, Pennsylvania

**Keywords:** HLA-B27, Immunomodulatory Therapy, Infliximab, TNF-α, Uveitis, Vasculitis

## Abstract

**Purpose:**

To determine the efficacy and safety of infliximab therapy in patients with HLA B-27-associated ocular inflammation resistant or intolerant to conventional immunomodulatory therapy.

**Methods:**

This was a retrospective observational case series. All cases were uveitic patients with positive HLA-B27, confirmed through HLA testing, resistant or intolerant to conventional immunomodulatory therapy. The primary outcome of the study was to identify the efficacy of infliximab determined by the control of inflammation, duration of remission, and the ability to reduce conventional immunomodulatory therapy. The secondary outcome was an improvement of two or more lines of best-corrected visual acuity (BCVA) on the Snellen visual acuity chart.

**Results:**

Twenty-four patients (38 eyes) were included in the study. All patients were followed for 24 months. Twenty-one (87.5%) patients completed 24 months of follow-up. Sixteen (66.7%) patients had active uveitis at the beginning of therapy. One patient out of these active patients had active inflammation at the end of follow-up period. Thirteen (87.5%) out of sixteen active patients were in steroid-free remission. The mean duration of treatment to induce remission was 16.5 months (range 6–24 months). Corticosteroid was stopped in 19 (90.5%) patients by the end of the study. At the end of the study, in patients who achieved remission, 14 (58.3%) patients were in remission on infliximab therapy and 6 (25%) patients were in remission off infliximab therapy. Of the 38 eyes, 8 (21.05%) showed improvement in BCVA (three eyes had successful cataract extraction with intraocular lens implantation during infliximab therapy with no subsequent inflammation), while 26 eyes (68.4%) had stable BCVA over the 24-month study period. The side effects included allergic reaction, fatigue, cellulitis, headache, restlessness, elevation of liver enzymes, and anemia. Two patients (*n* = 24, 8.3%) experienced severe adverse effects and the treatment was stopped prematurely in these two patients.

**Conclusion:**

Infliximab might induce and maintain the steroid-free remission in HLA-B27-associated ocular inflammation in patients resistant or intolerant to conventional immunomodulatory therapy.

##  INTRODUCTION

Acute anterior uveitis (AAU) is the most common form of intraocular inflammatory disease and can be associated with the human leukocyte antigen (HLA)-B27 haplotype in approximately 55–71% of patients.^[1,2]^ It usually manifests at a young age as recurrent, acute, non-granulomatous uveitis, with a unilateral or alternating bilateral presentation. The disease may involve posterior segment in the form of optic disc edema, macular edema, choroidal folds and effusions, exudative retinal detachment, and anterior and posterior scleritis.^[3]^ Intense inflammation and protein coagulum in the aqueous may lead to temporary or permanent severe visual impairment.

Topical corticosteroids, the first line of therapy, are sometimes insufficient to control the intraocular inflammation. Additional periocular and/or systemic corticosteroids and sometimes both are required to control the inflammation in more severe cases. Corticosteroid treatment increases the risk of systemic and ocular complications such as glaucoma and cataract. Therefore, corticosteroid-sparing conventional immunomodulatory therapies such as methotrexate, azathioprine, and mycophenolate mofetil (MMF) have been employed in the treatment of these patients. Biologic response modifier agents are the next step of the step ladder approach in the treatment of patients intolerant or resistant to conventional immunomodulatory therapy (IMT).^[4,5]^

Infliximab is a mouse–human chimeric immunoglobulin G1 (IgG1) monoclonal antibody that binds both the soluble and the membrane-bound precursor of tumor necrosis factor-alpha (TNF-α) and acts as a TNF-α inhibitor. It has been employed off-label in the treatment of non-infectious ocular inflammatory diseases refractory to conventional IMT with a high rate of inflammation control and few adverse effects.^[6,7,8,9,10,11,12,13,14]^

There are very few studies which have specifically focused on the HLA B-27-associated ocular inflammation.^[12,13]^ Even fewer studies have evaluated the efficacy and safety of infliximab therapy in patients with HLA-B27-associated ocular inflammation resistant or intolerant to conventional IMT.^[13]^ In this study, the efficacy and safety of steroid-free infliximab therapy were assessed in an HLA-B27 positive ocular inflammatory diseases in patients resistant or intolerant to conventional IMT.

##  METHODS

This study was a single-center retrospective observational case series. Approval for this study was obtained through the New England Institutional Review Board, which issued a waiver of informed consent, as this was a retrospective chart review analysis. This study was performed in accordance with the Declaration of Helsinki and was HIPAA compliant.

Electronic charts of patients with HLA-B27-associated ocular inflammation and infliximab (Remicade; Centocor, Inc, Malvern, Pennsylvania) therapy between July 2005 and October 2012 were examined. Those patients who received infliximab as the primary treatment were excluded.

Twenty-four patients (38 eyes) who had received at least one conventional IMT and/or another biologic response modifier agent were included in the study. The baseline demographic data, clinical diagnosis, and previous treatments were extracted from the charts. The best-corrected visual acuity (BCVA), slit lamp biomicroscopy, funduscopy positive findings, and intraocular pressure prior to and at 3, 6, 9, 12, 18, 24 months after starting the infliximab therapy were collected. Flare-ups, dose, and frequency of infliximab infusions, duration of follow-up, and severe adverse effects of infliximab were noted. Severe adverse effects were described as a severe side effect significant enough for the termination of infliximab therapy.

Standardization of Uveitis Nomenclature (SUN) Working Group grading scheme was used for the assessment of anterior chamber cell and flare.^[15]^ Cases seen prior to the publication of SUN guidelines were reclassified following the new anatomical classification system. Inactive anterior uveitis was defined as less than one plus cells in anterior chamber and inactive vitreous inflammation was defined as less than one plus vitreous haze.^[15,16]^ Inactive scleritis was defined based on Sen et al's study.^[17]^

In this study, refractory ocular inflammation was described as unresponsive inflammation or intolerance to a three-month course of treatment with at least two different conventional immunomodulatory agents.

Patients without inflammation for the consecutive six months of infliximab therapy were categorized as "remission on treatment" group. The "remission off treatment" group was defined as inflammation-free for a period of six consecutive months without infliximab treatment.

All patients had blood tests, which included a complete blood cell count (CBC), liver function tests (LFTs), and renal function tests before each infusion. They also had purified protein derivative skin test or QuantiFERON blood test, and anti-nuclear antibody (ANA) once at baseline and yearly afterward.

All patients received a loading dose of 5 mg/kg at 0 and 2 weeks, then every 4 weeks for five more infusions. However, some patients received higher doses of infliximab such as 7.5 mg/kg then 10 mg/kg after the third infusion because of the presence of inflammation in the complete ocular examination and/or ancillary tests. After six months, the frequency of infusions was stretched gradually from 6 to 12 weeks based on the response to the treatment and the absence of the active inflammation. The treatment was stopped once the patient was stable at the 12-week infliximab infusion intervals for four consecutive infusions.

In this study, the side effects were defined as adverse effects severe enough to justify the treatment termination. All patients responded to a questionnaire and the blood sample was collected to evaluate for possible medication toxicity in white blood cell (WBC), red blood cell (RBC), platelet (PLT), alanine aminotransferase (ALT), aspartate aminotransferase (AST), alkaline phosphatase (ALKP), gamma glutamine transferase (GGT), blood urea nitrogen (BUN), and creatinine (Cr) before each infusion.

The primary outcome of the study was to determine the efficacy of infliximab determined by the control of inflammation, duration of remission, and the ability to reduce conventional IMT. The safety of the study was defined as the absence of severe side effects. The secondary outcome was an improvement of two or more lines of BCVA on the Snellen acuity chart.

### Statistical Analysis

Data were presented as descriptive statistics (mean, percentages, range). Microsoft Excel 2010 (Microsoft, Richmond, VA) was used for statistical analysis. Paired and unpaired two-tailed *t*-tests were used to compare the number of medications, intraocular pressure, and visual acuity changes before and after the treatment. The level of significance was set at *P* ≤ 0.05.

##  RESULTS

Twenty-four patients (38 eyes) were included in the study. The average age of the patients was 44.7 ± 13.6 (12–67 years). Of the 24 patients, 14 (58.3%) were women and 14 (58.3%) had bilateral involvement. The prevalence of one or more associated systemic illness including ankylosing spondylitis, reactive arthritis, inflammatory bowel disease, psoriatic arthritis, and juvenile rheumatoid arthritis was 70.8% (17 of 24) (Table 1). Sixteen patients (*n* = 24, 66.7%) at baseline had active ocular inflammation, which was reduced to only one patient (*n* = 21, 4.7%) at the 24-month follow-up visit (Table 2). Of the sixteen patients with active ocular inflammation at baseline, 6 patients (37.5%) and 10 eyes (38.5%) had chronic inflammation, while the other 10 patients (62.5%) and 16 eyes (61.5%) were diagnosed with an acute flare up. All patients were on at least one conventional IMT and/or another biologic response modifier agent (Table 3).

**Table 1:** General characteristics and medication


**Characteristics**	**Value**	
Age – yr	
Mean ± SD	44.7 ± 13.6	
Range	12–67	
Female – no. (%)	14 (58.3)	
Systemic Diseases**${}^{†}$** – no. (%)	17 (70.8)	
AS	9 (37.5)	
AS and RA	2 (8.3)	
AS and IBD	1 (4.1)	
PA	2 (8.3)	
NSA	2 (8.3)	
JIA	1 (4.2)	
**Conventional therapy**	**Before infliximab patients (** ***n*** ** = 24) (%)**	**After infliximab patients (** ***n*** ** = 24) (%)**
Corticosteroids (by route)	
None	0 (0)	19 (79.1)
Topical	21 (87.5)	4 (16)
Periocular and/or intraocular${}^{¶}$	8 (33.3)	–
Oral and/or intravenous	13 (38.1)	3 (12.5)
IMT (by number of medications)**${}^{‡}$**	
None	0 (0)	7 (29.1)
1 medication	7 (29.2)	14 (58.3)
2 medications	8 (33.3)	2 (8.3)
≥3 medications	9 (37.5)	1 (4.1)
Side effects after infliximab (*n* = 24)	12 (50)
Allergic reaction	–	2 (8.3)
Fatigue	–	3 (12.5)
Cellulitis	–	2 (8.3)
Headache, restlessness	–	1 (4.2)
AST/ALT elevation	–	2 (8.3)
Anemia	–	2 (8.3)
**${}^{†}$**AS, ankylosing spondylitis; RA, reactive arthritis; IBD, inflammatory bowel disease; PA, psoriatic arthritis; NSA, nonspecific arthritis; JIA, juvenile inflammatory arthritis ${}^{¶}$At least one month before infliximab therapy **${}^{‡}$**IMT, Immunomodulatory therapy AST, aspartate aminotransferase; ALT, alanine aminotransferase		

**Table 2:** Inflammation status before and after the infliximab therapy excluding inactive patients


**Ocular inflammation**	**Before Infliximab [patients (%)] (***n***** =16)****	**After Infliximab [patients (%)] (***n***** = 16)****				
	**3 months**	**6 months**	**12 months**	**18 months**	**24 months**
Active	16 (100)	3 (16.6)	2 (12.5)	2 (12.5)	2 (12.5)	1 (6.25)
Anterior uveitis	11 (68.7)	1 (6.25)	–	–	1 (6.25)	–
Scleritis	2 (12.5)	–	–	–	–	–
Vitritis	1 (6.25)	–	–	–	–	–
Retinal vasculitis	–	1 (6.25)	1 (6.25)	1 (6.25)	1 (6.25)	1 (6.25)
Anterior uveitis with retinal vasculitis	–	–	1 (6.25)	–	–	–
Vitritis with retinal vasculitis	1 (6.25)	–	–	1 (6.25)	–	–
Papillitis with retinal vasculitis	–	1 (6.25)	–	–	–	–
Vitritis, papillitis, and retinal vasculitis	1 (6.25)	–	–	–	–	–

**Table 3:** Immunomulatory therapy before starting the infliximab therapy in patients with HLA-B27 ocular inflammation


**Immunomodulatory therapy**	**Number (%)**
Methotrexate	10 (41.6%)
Mycophenolate Mofetil	5 (20.8%)
Humira	5 (20.8%)
Cyclosporine	2 (8.33%)
Chlorambucil	1 (4.16%)
Daclizumab	1 (4.16%)
Etanercept	1 (4.16%)
Azathioprine	1 (4.16%)
Celecoxib	1 (4.16%)
None	4 (16.6%)

**Table 4:** Management of patients with ocular inflammation flare-up during the study period


**Study period**	**Number of patients with flare-up of ocular inflammation**	**Treatment for flare-up**
3-6 months	4 (17.4%, * n* = 23)	3 patients (* n* = 23, 13%) – reducing the infliximab infusion interval 1 patient (* n* = 23, 4.3%) – boosting infliximab dose 1 patient (* n* = 23, 4.3%) with persistent vasculitis – MMF and CSA were substituted for methotrexate and adalimumab
6–12 months	4 (19%, * n* = 21)	1 patient (* n* = 21, 4.8%) with papillitis, vitritis, and retinal vasculitis – received additional intravenous pulse steroids and was switched to cyclophosphamide 2 patients (* n* = 21, 9.5%) – received increased dose of infliximab 1 patient (* n* = 21, 4.8%) with retinal vasculitis – received increased doses of infliximab along with MMF and CSA
12–18 months	2 (9.5%, * n* = 21)	1 patient (* n* = 21, 4.8%) with persistent retinal vasculitis – received increased dose of infliximab along with oral corticosteroids 1 patients (* n* = 21, 4.8%) – relapsed with anterior uveitis and was restarted on infliximab
18–24 months	3 (14.3%, * n* = 21)	1 patient (* n* = 21, 4.8%) – added methotrexate 1 patient (* n* = 21, 4.8%) – reducing interval and boosting dose of infliximab 1 patient (* n* = 21, 4.8%) – increasing the dose of infliximab with oral steroids
MMF, mycophenolate mofetil; CSA, Cyclosporin-A		

**Figure 1:**
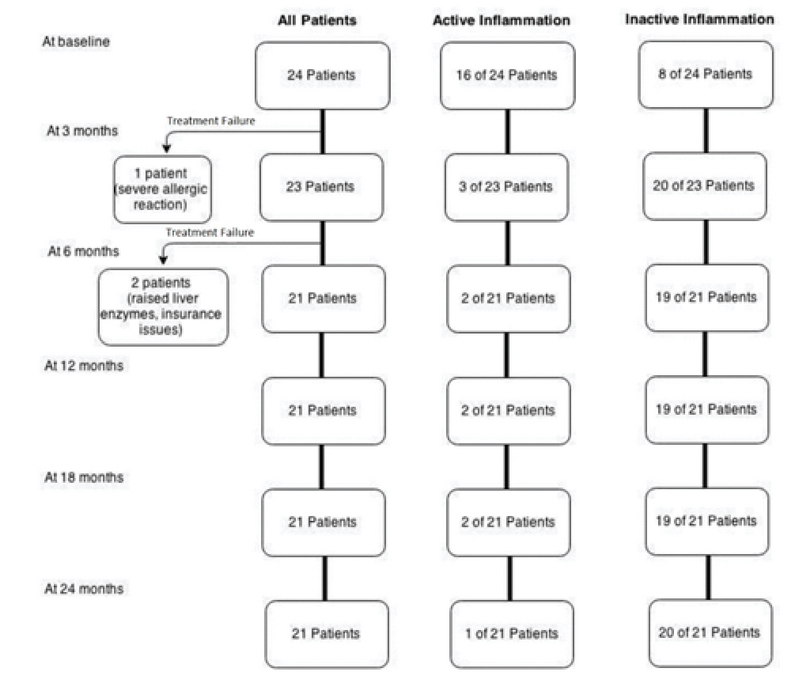
The number of patients in total, with active and inactive disease at each follow-up visits.

The infliximab therapy was prematurely stopped in three patients (12.5%) between the three- and six months follow-up period due to one patient with a severe allergic reaction (itching, rash, and shortness of breath during the infusion), one patient with a significant rise in liver enzymes (ALT and AST more than 10 times of the normal), and one patient with insurance coverage problems (Figure 1).

**Figure 2:**
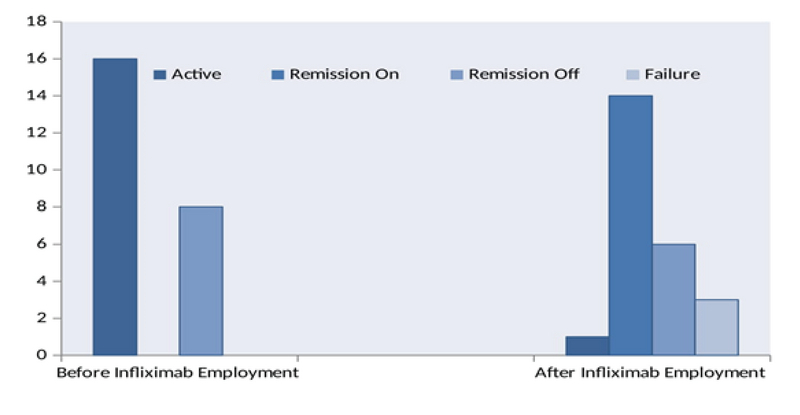
The number of patients with active disease, remission on infliximab Infliximab, remission off infliximab therapy before starting the treatment and at the end of the follow-up period.

Of the eight inactive patients (33.3%) at baseline, six patients (25%) were started on infliximab treatment due to intolerance to previous conventional IMT therapy. In the other two patients (8.3%), infliximab was started for an active systemic disease by their rheumatologist. The latter two developed an ocular inflammation during the first three months of follow-up.

For the patients with active disease at baseline, the duration of the treatment required to induce remission varied from less than six months in four (*n* = 16, 25%) patients to more than one year in five (*n* = 16, 31.3%) patients. Six patients (*n* =16, 37.5%) were in remission by the second year of the follow-up period. At the end of the study, 14 (58.3%) patients were in remission on infliximab therapy and six (25%) were in remission off infliximab therapy (Figure 2). The mean duration between starting the treatment and induction of remission was 16.5 months (range, 3–24 months). Table 4 demonstrates how patients with flare up were treated during the study period.

Before employing infliximab therapy, all patients had received one or more routes of corticosteroid administration, including topical, periocular, systemic therapy based on the severity of the disease. Twenty-one patients (87.5%) were receiving topical corticosteroids; six patients (37.5%), two patients (8.33%), and another two patients (8.33%) received transseptal, intravenous, and intravitreal corticosteroids, respectively. Eleven patients (45.8%) were on oral prednisone; higher doses (30–60 mg) were employed in four patients (16.7%) with severe active inflammation. Seven patients (29.1%) were on maintenance oral prednisone therapy with an average dose of 8.7 mg (range, 7–10). Nineteen patients (90.5%) discontinued corticosteroids by the end of the study. At the 24-month follow-up, 13 patients (87.5%) out of the 16 active patients were in steroid-free remission. During the 24 months of follow-up, the number of conventional IMT agents were reduced in 14 (58.3%) patients, the dosage of medications was halved in two patients (8.3%), and medications were discontinued in five (*n* = 21, 23.8%) patients (Table 1). The average number of medications before starting treatment was 2.3 ± 1.14 and at the end of the follow-up period was 0.08 ± 0.2. The difference in the average number of medications before starting the treatment and at the end of the study period was statistically significant (*P* < 0.0001).

Of the 38 eyes, 8 eyes (21.05%) showed improvement in BCVA (three eyes had cataract extraction with intraocular lens implantation during infliximab therapy with no subsequent eye inflammation), while 26 eyes (68.4%) had stable BCVA over the 24-month study period. The mean visual acuity before starting infliximab treatment was 20/30–20/40 (0.22 ± 0.43LogMAR) and was 20/25 (0.1 ± 0.24LogMAR) after the treatment. The mean difference of visual acuity before starting infliximab therapy and at the end of 24 months of follow-up was not statistically significant (*P* = 0.14). Moreover, the changes in visual acuity in each eye before starting the treatment and at the last follow-up visit were not statistically significant (*P* = 0.06).

The average intraocular pressure in all uveitic eyes of patients before starting infliximab therapy was 16.3 ± 4.56 mmHg (10–34). The averages of intraocular pressure in the worse and better eyes of patients were 18.2 ± 4.61 mmHg and 14.5 ± 3.44 mmHg, respectively. At the last follow-up visit, the average of intraocular pressure in all uveitic eyes of patients was 14.9 ± 2.00 mmHg (10–20). Averages of intraocular pressure in the worse eyes and better eyes of patients were 14.9 ± 2.07 and 14.57 ± 2.2 mmHg, respectively. Using the paired *t*-test, the difference between the intraocular pressure of the worse eye of each patient before starting the treatment and at the last follow-up was statistically significant (*P* = 0.004); however, this difference was not significant in the better involved eyes of each patient (*P* = 0.8).

The primary dose of infliximab was 5 mg/kg body in 19 patients. In three patients (12.5%), infliximab was started at the highest dose of 10 mg/kg: one patient was monocular, one had severe scleritis, and one had extensive retinal vasculitis.

The side effects included allergic reaction, fatigue, cellulitis, headache, restlessness, elevation of liver enzymes, and anemia. The treatment was discontinued in three (12.5%) patients; two due to the development of side effects and one due to insurance problems. These three patients were considered as treatment failures.

##  DISCUSSION

The association between HLA-B27 positivity and AAU with or without a spectrum of other inflammatory diseases was originally described in 1973 and is one of the strongest HLA-disease associations.^[18,19]^ TNF-α gene controls the production of TNF-α, which is a cytokine and inflammatory mediator in animal models of uveitis. TNF-α has been detected in the uvea and retina at the early stages of acute uveitis. In addition, TNF-α has been found in higher levels in the sera and aqueous humor of patients with uveitis.^[5,20]^ It may induce expression of chemokines, adhesion molecules, and other proinflammatory cytokines causing protracted inflammatory process.^[21]^ Its blockade is associated with decreased ocular inflammation in animal models.^[22,23,24]^ Sahrawi et al proposed that HLA B27-positive individuals show a higher susceptibility toward development of intraocular inflammation in the presence of an A allele at nucleotide-238 and, to a lesser degree, at nucleotide-308 of the TNF-α gene promoter.^[25]^ These findings formed the rationale for using the TNF-α inhibitor biologic agents in HLA-B27-associated ocular inflammatory diseases. Infliximab and adalimumab are the most common TNF-α inhibitor biologic response modifier agents employed in the field of ophthalmology. The successful use of infliximab and adalimumab for controlling ocular and systemic inflammatory conditions has been reported in different studies.^[26,27,28,29,30,31]^ Adalimumab is the only FDA-approved biologic for the treatment of noninfectious uveitis.^[29,30,31]^

The median age (44 years) of our study sample and the prevalence of associated systemic illness (70%) matched earlier studies.^[27][28][29,30,31]^ Infliximab was well-tolerated and BCVA was improved or remained stable in the majority of our patients. Previous studies reported the success of infliximab therapy in different ocular inflammatory diseases with various etiologies including HLA-B27-associated uveitis.^[29,30,31][32]^ In two studies by Foeldvari et al and Suhler et al, the success of infliximab in JIA-associated uveitis and panuveitis, intermediate uveitis and posterior uveitis was 70% and 78%, respectively.^[28,31]^ Kim et al and El-shabrawi et al found that infliximab (3–5 mg/Kg body weight) was effective in 76.2% and 66.7% of patients with HLA-B27 anterior uveitis.^[33,34]^ In our study, ocular inflammation was controlled in 83.3% of patients at the end of two years; higher than that reported in previous studies.^[29][32][33,34]^ This can be attributed to adjustment of infliximab dose up to 10 mg/kg based on the response to infliximab therapy.^[28,31][32][33]^ While previous studies used pulse or oral corticosteroid therapies to control acute inflammation, the aim of our study was to achieve steroid-free remission. Thus, a significant number of our patients were not on steroids for their ocular inflammation at the 24-month follow-up visit. This can be interpreted by the higher doses of infliximab and longer duration of treatment that we employed in our patients.^[14,35]^

The starting dose for Infliximab therapy was 5 mg/kg in all patients except those with more severe involvement such as retinal vasculitis and scleritis. We followed the protocol of two loading doses and five infusions every four weeks before stretching the infusions. Slow tapering of the infusions has been proved as an effective way to maintain remission in patients on anti-TNF-α inhibitor therapy.^[34,36,37,38,39]^ This was also demonstrated in our study. Some of our patients needed to increase the dose of infliximab, which may be due to three possible hypotheses. The first hypothesis is the development of an antibody against the infliximab molecule due to its murine constituent. This may be prevented by adding a low-dose anti-metabolite to the regimen; however, we were unable to make this conclusion due to low sample size. A second possible reason to increase the dose of infliximab is tachyphylaxis to the medication itself. The third mechanism is the lower dose of medication per age in younger individuals due to their growth spurt and an increase in their weight.

In this study, there were two patients with HLA-B27 associated active systemic inflammation and quiet eyes who were started on infliximab by the rheumatologists and their intraocular inflammation occurred early in the course of infliximab therapy. There are two explanations for this finding. First, it might be the natural course of the HLA-B27-associated anterior uveitis, which was not under control with the previous regimen and the time for infliximab to control the inflammation was not sufficient. Second, in our experience, there are some patients with HLA-B27-associated ocular inflammation who need higher doses of infliximab for controlling ocular inflammation. In these patients, ocular inflammation can recur despite their systemic symptoms being controlled with a standard dose of infliximab. That is why the dose adjustment for ocular inflammation control might be necessary after the third infliximab infusion.

Our study had the inherent limitations of a retrospective study design with a small sample size. As a tertiary care clinic, a large proportion of our patients present with severe, long-standing, and often unresponsive disease. Moreover, due to the nature of our referral center which has more severe and complicated cases, the trend is toward a more aggressive therapy such as infliximab rather than a less aggressive treatment such as adalimumab injection, and this can also be a source of selection bias. Furthermore, similar to other retrospective studies, the inability to control for confounding factors such as changes in topical, local, and systemic corticosteroids and IMTs for eye and systemic conditions may allow for the development of uveitis early in the course of infliximab therapy.

In summary, Infliximab therapy might be an effective and safe method of treatment for inducing and sustaining the steroid-free remission in patients with HLA-B27-associated ocular inflammation, which can be a serious and potentially blinding condition if left untreated or incompletely treated. However, to validate these findings, more potent studies are required.

### Disclosure Statement

Dr. C Stephen Foster declares the following:

Consultancies with Aldeyra Therapeutics (Lexington, MA), Allakos (Redwood City, CA), Bausch & Lomb Surgical, Inc (Rancho Cucamonga, CA), Eyegate Pharma (Waltham, MA), Genentech (South San Francisco, CA), Novartis (Cambridge, MA), pSivida (Watertown, MA). Grants or grants pending with Aciont (Salt Lake City, UT), Alcon (Aliso Viejo, CA), Aldeyra Therapeutics (Lexington, MA), Bausch & Lomb (Rochester, NY), Clearside Biomedical (Alpharetta, GA), Dompé pharmaceutical (Milan, Italy), Eyegate Pharma (Waltham, MA), Mallinckrodt pharmaceuticals (Staines-upon-Thames, UK), Novartis Pharmaceuticals (Cambridge, MA), pSivida (Watertown, MA), Santen (Osaka, Japan).

Payment for lectures including service on speaking bureaus: Alcon (Aliso Viejo, CA), Allergan (Dublin, Ireland), Mallinckrodt pharmaceuticals (Staines-upon-Thames, UK).

Stock or Stock Options: Eyegate Pharma (Waltham, MA)

Other authors have nothing to declare.

### Data Availability Statement

The data that support the findings of this study are available from the corresponding author, [CSF], upon reasonable request.

##  Financial Support and Sponsorship

None.

##  Conflicts of Interest

There are no conflicts of interest.
